# ‘We All of Us Make Mistakes’: Medical Negligence in Interwar General Practice

**DOI:** 10.1093/shm/hkae097

**Published:** 2025-01-27

**Authors:** Anne Hanley

**Affiliations:** Senior Research Fellow and UKRI Future Leaders Fellow, Department of Applied Health Sciences, College of Medicine and Health, University of Birmingham, Birmingham B15 2TT, UK

**Keywords:** negligence, ethics, general practice, syphilis, microhistory

## Abstract

In August 1920, Dr Lysander Maybury began a course of weekly injections into the longitudinal sinus of newborn Leslie Shewry. Although Maybury told Leslie’s parents that he would be giving their son injections, he did not tell them that he had diagnosed congenital syphilis. The precise nature of Leslie’s treatment was also unknown to his parents until many years later when they brought a case for damages against Maybury. They alleged that he had wrongly diagnosed and unnecessarily and improperly treated their son, leaving him permanently disabled. Furthermore, they alleged that he lacked the necessary skills and training to perform such delicate injections and that he was negligent in persisting with treatment when he knew that Leslie suffered convulsions after each injection. Shewry v. Maybury is a microhistory in which the intimate disruptions wrought by one man reveal a great deal about the nature and consequences of medical negligence in interwar Britain.

On 20 July 1920, Leslie Shewry was born in Southsea to Sergeant Marcus Shewry of the Portsmouth Borough Police Force and his second wife, Charlotte. Ten days later, Dr Lysander Maybury, the general practitioner (GP) who had attended Leslie’s birth, diagnosed him with jaundice.^1^ On 3 August, Maybury commenced a course of weekly injections of ‘a liquid … in the neighbourhood of the brain’.^2^ It later transpired that this mystery liquid was novarsenobilion (NAB)—a treatment for syphilis. But the Shewrys claimed that its precise nature had been unknown to them until they began legal proceedings against Maybury several years later. Maybury had not informed them that he had diagnosed or treated their infant son for congenital syphilis.^3^

After an adjournment of several months when Shewry, now an Inspector, was being treated for lung cancer, the case finally began on 11 June 1929 before the High Court of Justice, King’s Bench Division.^4^ Shewry alleged that Maybury had wrongly diagnosed and unnecessarily and improperly treated Leslie for congenital syphilis throughout August 1920, leaving him ‘permanently disabled, both mentally and physically’. The writ issued against Maybury in June 1928 claimed that Leslie had ‘sustained, as a result of his treatment, thrombosis of the superior longitudinal sinus’—the vessel responsible for draining blood from the lateral aspects of the anterior cerebral hemispheres.^5^ It also alleged that Maybury ‘had not sufficient skill or training’ for such injections and was negligent in persisting with treatment when he knew Leslie suffered convulsions after each injection.^6^

At first glance, Shewry v. Maybury was a straightforward case of repeated failings by a single individual. But on closer inspection, we see a medical culture that excused and even enabled Maybury’s mistakes and actions. It is a microhistory in which the intimate disruptions wrought by one man reveal a great deal about the nature and consequences of medical negligence in the early twentieth century.^7^ By contextualising Leslie’s treatment, the Shewrys’ search for restitution and Maybury’s efforts to avoid accountability, we realise that this case was both singular and a symptom of wider institutional malaise. Certainly, some doctors disagreed with Maybury’s actions and even publicly disavowed them. The jury’s verdict was that he had been negligent, both in his diagnosis and general treatment. And as we shall see, this was not the first case in which he was found to have behaved negligently. Yet despite all this, the medical profession closed ranks, denied the validity of his patients’ experiences, sought to credit Maybury with expertise he did not possess and protected him from the consequences of what seems to have been a pattern of misconduct.

The study of medical harm and iatrogenic injury is increasingly popular, prompted in part by various present-day revelations about systemic healthcare failures.^8^ Then as now, it appears that harm was rarely intentional, more often resulting from error, overwork, inurement, carelessness or hubris.^9^ As one doctor put it when asked about Maybury’s actions, ‘we all of us make mistakes. Now having heard the evidence in this case, I think he acted carelessly’.^10^ Harm was compounded by a wider medical culture that was itself sustained by ingrained attitudes, ideologies and behaviours at an individual and collective level. Rarely have historians turned their attention to harm in general practice, focussing instead on institutions.^11^ By the 1920s, consulting one’s GP was more common than seeking institutional care. Yet the records of these consultations are far scarcer, making cases like Leslie’s all the more important.

Beyond the few cases that occasioned legal proceedings, press coverage or write-ups in medico-legal texts, we know little about the scale of historical medical harm and even less about its impact on patients and their families. Before the 1957 Bolam decision, which redefined the standards by which medical negligence was judged, doctors were theoretically more vulnerable to legal action.^12^ But prospective plaintiffs still faced considerable financial and social hurdles, making legal redress accessible to only a minority. The complaints that made it to court were only the tip of an indeterminately large iceberg.^13^

Shewry v. Maybury is therefore a rare exception. But even here, the records are fragmentary. They are mostly defence-witness statements and court notes, as well as letters from Maybury to Dr Felix Eberlie, a key witness who was Maybury’s assistant when the alleged harm took place. Apart from Shewry’s sworn statement and a small collection of press reports, we have only the evidence compiled by Maybury and his team, who took great care to craft a narrative that presented him and his clinical actions in the most favourable light. We do not have Eberlie’s replies, nor any correspondence sent to Maybury by the many medical men he sought as witnesses. Nonetheless, these records are an extraordinary microhistory of the attitudes and actions of a medical man accused of negligence. And through these records, we also glimpse the wider professional networks and power structures that shaped interwar medicine.

Despite referring regularly to intimate and distressing aspects of Leslie’s health, the records remain unredacted and publicly available. The names of those involved even form part of Wellcome’s archival references. The public nature of these records adds another layer to the already complex questions we must ask about our ethical responsibilities to the dead and their descendants.^14^

Historians who choose not to pseudonymise or anonymise contend that these processes obliterate for a second time the identity and agency of those already marginalised in historical records. In the history of medicine, it risks perpetuating the reduction of an individual to a collection of pathologies. But are we inflicting harm when we endeavour to ‘rescue’ them from what E.P. Thompson famously described as ‘the enormous condescension of posterity’?^15^ Even with the best intentions, we risk rendering them two-dimensional tools for accessing the past.^16^ Certainly, the deceased cannot consent to becoming subjects of historical scrutiny. But neither have they consented to being rendered anonymous.

Pseudonymisation and anonymisation also protect the posthumous reputations of those who caused and enabled harm. During his short life, Leslie was subjected to invasive and distressing treatments for which his parents were denied the opportunity for consent. Subsequently, the family made the difficult and costly decision to seek public redress. In that process, the medical men to whom they had turned in 1920 for help attempted to rewrite, minimise and obscure the Shewrys’ experiences to protect their own reputations. So much of the Shewrys’ experience was defined by the absence of consent and autonomy. To pseudonymise or anonymise their story now would silence them a second time and shield those whose actions compounded their distress.

As historians, we are uniquely placed to meet such challenges. But it can never be a one-size-fits-all approach. Rather, it requires what Richard McKay describes as ‘caring pragmatism’—a continually evolving process grounded in universal ethical principles and responsive to the specific sensitivities of any given project.^17^ It is especially helpful for building responsible, respectful and empathetic engagement with traumatic pasts. We cannot avoid being affected by the traumatic events we research.^18^ But balanced against this is the need for professional detachment.^19^ For my own part, I wrote this article in the months just before and after my own son was born. The experience was both deeply empathetic and determinedly analytical. We cannot allow our empathy and positionality to weaken our analysis or prejudice us against those whose actions caused distress. In critiquing Maybury’s clinical practice and his subsequent efforts to control the narrative around that practice, this article reflects the difficult methodological and emotional path that historians must navigate through the archives.

## Diagnosis

There is a long-standing debate about the extent to which we can or should impose retrospective diagnoses.^20^ In most cases, we simply do not have enough data about the individual, their society or the cultural meanings that their society attached to different disease categories. We do not know their full range of symptoms or are reliant on the incomplete and subjective records compiled by care givers. Rarely can we turn to records of laboratory analyses. And even if we could, would these tell us anything meaningful about an individual’s experiences? As Chris Millard puts it, in the act of retrospective diagnosis, we risk ‘projecting currently valid knowledge back through time’.^21^ But this article is not a pathography of Leslie. While it may not be possible, meaningful or ethical to attempt a diagnosis, we have enough information to question the illness narrative compiled by his doctors. We can scrutinise Maybury’s diagnosis according to the knowledge and technologies available in 1920 and compare his clinical actions against the established standards of safe, ethical venereological care.

Medicine has become organised according to what Charles Rosenberg describes as ‘a vocabulary of disease pictures’ underpinned by aggregated clinical findings and agreed symptomatology.^22^ Standardised disease pictures, now so fundamental to our illness experiences, helped to rationalise and legitimate clinical decision-making.^23^ It was the basis of Maybury’s defence, which focussed on three claims: he had correctly diagnosed Leslie; his treatment for congenital syphilis was, therefore, appropriate; and the treatment had been properly and skilfully administered.^24^ But as preparations for the court case progressed, it became increasingly clear that his grasp of syphilis’s symptomatology had been limited.

While preparing his defence, Maybury claimed that, soon after birth, Leslie’s skin became ‘inflamed over the napkin area, buttocks, testicles, penis, lower parts of the abdomen, mucous tubercles around the anus, mucous tubercles and fissures in the anus, that as the rash healed it became scaly, that the child has snuffles and it did not thrive’.^25^ This litany of rapid-onset symptoms departed from Charlotte Shewry’s assertion that Leslie was not unwell until the injections and then only developed neurological complications.^26^ Maybury’s account was also at odds with the established disease picture of congenital syphilis that had been refined throughout the nineteenth and early twentieth centuries in the pages of the medical press and numerous treatises.

It was widely understood as early as the 1870s that signs of congenital syphilis were often absent at birth, appearing in the weeks afterwards.^27^ Maybury claimed that Leslie’s jaundice was indicative of syphilis, but jaundice is a common condition among otherwise healthy newborns. If an infant presented with a suspected syphilitic rash, doctors should expect the palms of the hands and soles of the feet to be affected.^28^ Yet according to Maybury, Leslie’s rash did not extend beyond his lower torso. Equally unusual was Maybury’s description of the rash as scaly. In 1896, J.A. Coutts had noted that moisture around an infant’s buttocks caused the scales of a desquamating syphilitic rash to soak off, leaving the skin ‘raw or brightly glistening’.^29^ When a rash remained scaly, Hurbert Armstrong, physician to the Liverpool Infirmary for Children, was inclined to diagnose seborrhoeic eczema.^30^ Thomas Ballard went even further, asserting in 1874 that many infantile genito-anal rashes were not pathological at all, but the natural irritation from constant contact with urine and faeces.^31^ Although it was generally assumed that a congenitally syphilitic rash would manifest around an infant’s genitals and anus, doctors were increasingly questioning the logic of this assumption. Ballard scorned what he viewed as ‘a strange perversion of reasoning … as if it were inferred that because adults communicate venereal disease to each other by contact of the genital organs, therefore the disease must have a natural habitat in those regions’ among infants.^32^ This view was reiterated by J.E.R. McDonagh in 1920 when he stressed that ‘a rash on the buttocks is not absolutely diagnostic of syphilis, being more usually due to the use of dirty napkins’.^33^

Missing from Maybury’s long list of symptoms were some of the most classic indicators of congenital syphilis in infants: pot belly, withered skin and ‘the prematurely aged and wrinkled face’. These, according to the dermatologist Pernet George, were ‘so characteristic that once observed they are not readily forgotten’.^34^ By the late-nineteenth century, it was also generally believed that newborns died ‘speedily’ if they were so afflicted by congenital syphilis that neurological and physical symptoms manifested soon after birth.^35^ Yet Leslie lived for many years. Even as he grew, he did not develop any of the classic manifestations that could reasonably be expected following a severe newborn affliction of the type described by Maybury.^36^ The symptomatology of Leslie’s supposed infection was so unusual that Harold Burrows, surgeon to the Royal Portsmouth Hospital, doubted whether Maybury ‘ever saw in this child the stigmata of syphilis’.^37^

Importantly, each of Leslie’s Wassermann tests were negative. Developed in 1906 by August Paul von Wassermann, this complement-fixation test used samples of blood or cerebrospinal fluid to check for the presence of antibodies specific to patients with syphilis. Samples were collected by the attending doctor and sent to a local laboratory for analysis by a qualified pathologist, who then produced a report for the doctor.^38^ By 1920, when Leslie’s blood was first sampled, Britain’s Medical Research Committee (MRC) had standardised the processes for Wassermann testing, resulting in far greater reliability.^39^ While recognising that the reaction was not yet perfect, the *Lancet* praised the significant developments outlined in the scientific report submitted to the MRC in 1919 by Paul Fildes and R.J.G. Parnell, who found that ‘if a test is reported negative … it will be more or less improbable that the case is one of syphilis’.^40^ Acknowledging that human and technical error could not be completely avoided, the MRC was nonetheless adamant that ‘there is no process of biochemical diagnosis that gives more trustworthy information or is liable to a smaller margin of error’.^41^ This was reiterated by Professor Hubert Turnbull of the London Hospital’s Pathological Institute. Having been commissioned by the MRC to evaluate the test’s accuracy, Turnbull concluded in 1920 that it was ‘a diagnostic weapon of astonishing precision’.^42^

Leslie’s consistently negative results became a problem for Maybury’s defence. He confided to Eberlie in 1928 that ‘I am not labouring in my defence that in 1920 the WR was very uncertain because at that time the Ministry of Health under the VD Scheme had standardised the methods’.^43^ If he was aware of the Wassermann test’s improved reliability when he diagnosed Leslie in 1920, why did he ignore a negative result? If, on the other hand, he was unaware of these developments, it suggests that he was using a new technology without fully understanding it. Even before the case went to court, Maybury recognised that his diagnosis was questionable, conceding to Eberlie that his solicitor ‘will advise not stressing syphilis’.^44^ And under cross-examination he even admitted that his diagnosis may have been wrong.^45^

Yet his legal team proceeded to call several witnesses to testify that the reaction had been too unreliable to trust without reference to a patient’s physical condition. Richard Macpherson, Medical Superintendent of the Portsmouth Infirmary, had tested Leslie’s blood and cerebrospinal fluid. Again, the results were negative. But like Maybury, Macpherson was adamant that this did not alter his opinion that Leslie was congenitally syphilitic.^46^ It should be noted, however, that Macpherson reported having under his care several dozen children with ‘practically identical’ symptoms but who had never been treated with mercury or NAB, presumably because they did not have syphilis. In his efforts to prove Maybury’s competency, Macpherson had inadvertently demonstrated that Leslie’s symptoms could have been caused by conditions other than congenital syphilis.^47^ Indeed, in a bizarre twist, Macpherson testified that Leslie actually had ‘a typical case of Little’s disease’.^48^ Yet despite all this, Maybury spent considerable time crafting explanations for how Leslie might have been syphilitic while returning consistently negative results.^49^

Certainly, Maybury and Macpherson were not alone in questioning the reaction’s reliability.^50^ The more cautious or sceptical among the medical profession expressed what Rosenberg described as ‘the fear that a brash and burgeoning scientific medicine meant … excessive dependence on the laboratory’s tools and findings’, potentially cheapening doctors’ ‘holistic and intuitive skills’.^51^ Writing in the *BMJ* in 1920, Charles F. Marshall and Ernest G. French advised that ‘in cases in which the laboratory findings are negative, it is better practice to reply upon one’s clinical experience and put the patient under treatment if the clinical signs are suggestive of syphilis’.^52^ The following year, Hugh Wansey Bayly, pathologist to the London Lock Hospital, also warned against relying on Wassermann results in cases of suspected congenital syphilis without first establishing whether the parents were syphilitic.^53^ But Maybury failed to take a family history. Instead, he fixated on ambiguous symptoms, attributing them to a disease that had long been known as ‘the great imitator’ of many other conditions.^54^

Despite his keenness to employ the latest technologies, Maybury displayed a fundamental uneasiness with them. The rapidity and complexity of technological change meant that even respected venereologists sometimes struggled to keep pace. In 1911, James Ernest Lane, surgeon to the London Lock Hospital, openly admitted that ‘as regards the Wassermann test, I am compelled to rely on the investigations of those who are fortunate enough to have entered this work and this profession considerably later than I did’.^55^ Shewry v. Maybury is a good example of the impact of such generational lags on patient wellbeing, demonstrating how a doctor responded when confronted by the realisation that he was being technologically outpaced.

Despite claiming that ‘all the syphilitic men’ agreed with him, Maybury was clearly disappointed by McDonagh’s assertion that ‘an infant with undoubted signs of congenital syphilis invariably gives a positive Wassermann reaction’.^56^ McDonagh had long held this view, stating in the *Journal of the Royal Society of Medicine* as early as 1910 that ‘congenital syphilitics tended to give a strong positive reaction, which remained so throughout life’.^57^ If, as Maybury claimed, Leslie had indisputable symptoms of congenital syphilis, his Wassermann test should have been positive. That McDonagh, a sceptic of Wassermann testing, was adamant that infected infants returned reliable positive reactions, raised further questions about Maybury’s diagnosis.^58^ Equally disappointing for Maybury was Fildes’s professional opinion of Leslie’s case, prompting him to concluded scornfully that ‘all pathologists swear by the accuracy of the WR. They are Popish in their infallibility …. [I]t is difficult to find a pathologist who is also conversant with the clinical signs of syphilis’.^59^ Yet it was Fildes’s expertise in Wassermann testing as well as syphilis’s pathology and symptomatology that had made him an appealing prospective defence witness.

Having probably misdiagnosed Leslie, Maybury went to considerable lengths to construct a narrative that did far more than just rationalise or minimise his mistake. He presented his original diagnosis as the only reasonable one at which an experienced doctor might arrive. His actions aptly illustrate Rosenberg’s observation that diagnosis ‘helps constitute and legitimate the reality that it discerns’.^60^ Maybury’s case hinged, not on the accuracy of his diagnosis, but on whether his subsequent treatment had caused irreversible harm. Yet, in the face of mounting evidence, he still directed enormous time and energy to maintaining the rightness of his diagnosis because it legitimated his subsequent actions and preserved his professional reputation and credibility.

## Treatment

Maybury and Eberlie’s accounts of Leslie’s treatment were inconsistent. They produced only partial records, claiming not to recall specifics. At the same time, they insisted that every congenitally syphilitic child had been diagnosed and treated correctly and showed marked improvement.^61^ In his written statement, Eberlie was adamant that ‘the infants we injected with NAB all showed improvement; I do not remember any infant which showed any ill effects following NAB injection into the longitudinal sinus’. However, he could not remember Leslie’s specific condition or whether he suffered convulsions following each injection.^62^ Eberlie also insisted that ‘there was no negligence in making the injections’.^63^ But he had not assisted at all of Leslie’s injections. As Maybury’s own solicitor wrote to Eberlie, ‘you are unable to recollect the precise case and can only speak in general terms as to Dr Maybury’s technique and the care exercised by him in cases such as this’.^64^

In its post-trial defence of Maybury, the *BMJ* criticised the statute of limitations governing cases of alleged negligence against children, claiming that ‘it was not possible after many years fairly to bring to light all the relevant materials in a condition in which they might be suitably marshalled and impartially examined’.^65^ The journal pressed its readers: ‘what were *you* doing on a given date in 1920? How impossible it was to recollect! And this case depended not only upon recollection, but upon controversial recollection—recollection developed, perhaps during months and years of disappointment and resentment’.^66^ Such rhetoric cast aspersions on the Shewrys’ testimony while sidestepping whether Maybury had misremembered or misrepresented Leslie’s case history. Also missing from the *BMJ’s* impassioned defence was any acknowledgement that doctors were expected to keep detailed case notes. Indeed, the *BMJ* had attached great importance to this very practice only a few years earlier: ‘If there is not time to take notes, there is not time to see patients properly and something is at fault … Without the correction supplied by such records of fact, ill-founded opinions are apt to grow in a man’s mind and to prejudice his views’.^67^

As Maybury’s legal preparations progressed, it became clear that his notetaking in 1920 had been limited. The few notes he possessed had been compiled by Eberlie.^68^ Maybury denied knowing that Leslie suffered convulsions after his injections, claiming to have neither any relevant notes nor any recollection of having been informed.^69^ Yet Ada Helyer, a maternity nurse who had been instructed by Maybury to watch Leslie after his injections, testified that she informed Maybury that ‘within two hours, the baby had a fit and vomited’.^70^

Having insisted that there were no convulsions or that, if there were, he was unaware of them, Maybury spent considerable time crafting explanations for why congenital syphilis might cause ‘a twitch or convulsion’.^71^ Missing from these explanations was the possibility that Leslie’s convulsions might have resulted from unnecessary, inexpertly administered injections. He claimed that he could not have missed the vein because he had drawn blood into the syringe from Leslie’s longitudinal sinus before injecting NAB.^72^ But Burrows testified that ‘the vein into which the injection should be made was not visible, and a needle might very easily be thrust through it and so injure the tissues’.^73^ As several venereologists also cautioned, it was too easy for a vein to be missed and the injection to enter the surrounding tissue with serious or even fatal consequences.^74^

Maybury’s decision to intervene surgically was not in itself unusual. Before the National Health Service, surgery was a core aspect of general practice. Julian Tudor Hart estimates that in the late 1930s GPs were still performing an average of three operations a week.^75^ But in 1920, arsenical therapies like NAB were very new and difficult to administer safely. Salvarsan had only been developed in 1908 and neosalvarsan in 1911.^76^ Writing in 1917, Colonel Lawrence Harrison, who would soon become the new Ministry of Health’s VD advisor, reflected that ‘the precision demanded by the operation and the odd chance that the patient may display alarming symptoms immediately afterwards are deterrents to the practitioner carrying out these injections in his private consulting room’.^77^ The drugs themselves and their specialised apparatus were expensive. Their administration was also thought to require two specially trained assistants and a level of skill and experience beyond that of the average GP.^78^ According to Fildes and James McIntosh, administration might seem simple but the potential for accident and injury meant that doctors should consider their use ‘a heroic measure’.^79^ And as far as Lane was concerned, ‘all heroic measures … always imply danger to the patient’.^80^ In 1920, their administration was still so difficult that even Morna Rawlins, as Medical Officer in charge of the Female VD Department at Guy’s Hospital and gynaecologist to the Female Lock Hospital, confessed ‘a certain amount of fear for the arsenical preparation’.^81^

More common side effects ranged from high temperatures and chills, through to vomiting, rigour, swelling and pain. But more seriously, these drugs could cause necrosis, nerve damage, thrombosis, haemorrhagic encephalopathy and other ‘severe cerebral symptoms’ including convulsions.^82^ Although such side effects were uncommon, they were serious enough for specialists to caution against using arsenobenzenes without specialist training and, if they were used, to recommend medical observation for 24 hours after each injection.^83^

The required skill, expense, equipment and personnel presented obstacles for most doctors practising beyond larger hospitals or the emerging network of specialist VD clinics.^84^ These limitations were not unique to venereology. General practice was woefully under-equipped to deal with most types of specialist examination and treatment.^85^ But there were no regulatory mechanisms to stop GPs experimenting with new drugs. Appearing on Maybury’s behalf, Portsmouth’s Medical Officer of Health (MOH) testified that NAB had been supplied since 1917 to local doctors ‘regarded as capable of administering it properly, and Dr Maybury, who had the necessary qualifications, was one of the first to whom the preparation was supplied’.^86^ Maybury might have been qualified but did he possess the skill and facilities? C.R. Crawford, who practiced alongside Maybury in Southsea and had diagnosed Leslie with ‘epilepsy following injury to the brain’, insisted that GPs were not equipped to perform an operation ‘of such great difficulty’.^87^ But even if they were, he ‘would most certainly not feel justified in injecting NAB into the longitudinal sinus of an infant unless he had a positive result from the blood test’.^88^

According to his records, Maybury was the only person to attend three of Leslie’s five injections. On the other two occasions, he was joined by his eldest son, who was listed in the records as Monti. Still a medical student, Monti was not specially trained. Neither was Maybury’s dispenser, John Lane (formerly of the Naval Sick Bay), who had been present at only one injection but did not actively assist.^89^ Despite Maybury’s claims that Eberlie assisted at every injection and ‘keenly watched the operation’, his presence was recorded only once.^90^ Throughout the court case, Maybury’s competence and judgement were called into question. He attempted several of the injections single-handedly. He relied on the assistance of his unqualified son. And he disregarded the medical observations made by the nurse responsible for Leslie’s post-treatment care. These decisions, all of which went against the recommended standards of his day, indicate either over-confidence or a lack of familiarity with the safety protocols emerging around these drugs.

By 1920, GPs were urged to relinquish control of their more complex venereal cases to local VD clinics, where patients would receive specialist care that was free at the point of use.^91^ It was part of a wider culture of referral that had been emerging in general practice since the late-nineteenth century.^92^ As early as 1918 there were calls for GPs to ‘take the fullest advantage of the facilities afforded by the public venereal centres, both in diagnosis, in treatment in the case of individual patients, and for gaining experience himself in the administration of the newer remedies’.^93^ Of course, patients continued to be seen privately, even after the establishment of the National Health Service.^94^ But many GPs were redirecting patients to the VD Service.^95^ Some did so because they did not want to treat a distasteful class of diseases. Others, because they were uncertain of their diagnoses or because they recognised that new standards of venereological care were beyond them.

Why then, did Maybury not refer Leslie to the local VD clinic? He was certainly aware of its facilities.^96^ An important consideration was that referral would mean losing clinical and financial control of his patient.^97^ Indeed, the Shewrys’ legal team cast aspersions on Maybury’s financial motives, insinuating that he was guilty not only of negligence, but also self-interest. As the *BMJ* put it, ‘a new and terrible suggestion had been made, that this defendant never diagnosed congenital syphilis at all, and entered upon a series of operations described as hazardous or dangerous deliberately in order to put fees into his pocket’.^98^ The suggestion was so serious that during the proceedings, Hewart had to issue a stern warning against making unfounded allegations.^99^

But it cannot be denied that Maybury’s fees were substantial. Before the First World War, a single dose of salvarsan cost between 7s. and 10s., putting it beyond even the budgets of many institutions.^100^ By 1930, pharmaceutical manufacturers like Boroughs Wellcome & Co were selling a dozen 0.1gm phials of Kharsivan for 24s. Even their most expensive concentration—a dozen 0.6gm phials at 120s.—cost only 10s. per phial.^101^ Moreover, doctors like Maybury who were issued batches of NAB by their Local Authority as part of the Joint VD Scheme would have received these drugs free of charge or at a substantially reduced rate.^102^ Yet in 1920, Maybury had been charging 5 guineas (105s.) per injection.^103^ For a family on a police sergeant’s salary, the financial burden was enormous.^104^ When Shewry expressed concern about the expense and questioned why the injections were necessary, Maybury became defensive. According to Shewry, he made the exaggerated claim that ‘he had given the child a treatment that in Harley Street would have cost 500 guineas’, insisting that Leslie would die without it and if the Shewrys could not afford his fees, there were other patients who could.^105^ Even accounting for several years of inflation and the economic upheaval of a World War, Maybury’s fees were prohibitive. While some GPs were deterred by the expense and difficulty, others like Maybury saw opportunity. Indeed, it had long been acknowledged that VD treatments were a ‘lucrative item in the practices of many doctors, particularly in seafaring areas’ like Portsmouth.^106^ And all this unfolded against a backdrop of wider concerns about desperate VD patients at risk of exploitation by unscrupulous doctors—qualified and quack alike—prescribing ineffective, expensive remedies.^107^

In retaining control of his patients, Maybury also demonstrated reluctance to acknowledge that he needed help. He resented what he saw as interference from other health professionals. As evidenced during a lecture in Portsmouth by Marie Stopes in 1923, he was also parochial over matters that he believed were the preserve of doctors—in this case, birth control. In his view, ‘it should be left entirely to the medical profession and not dealt with by lay persons’.^108^ But it seems that he would not deal with birth control either, believing it to be unnecessary and harmful to familial happiness. In what *The Evening News* described as ‘the one discordant note in the meeting’, Maybury vehemently objected to Stopes’s argument that birth control protected women’s health because in his personal experience as a medical officer, ‘the largest families were the happiest’.^109^

This was not the only time that Maybury’s actions had been reported on unfavourably. In 1925 another patient had successfully sued Maybury for the enormous sum of £1000, alleging, as the *BMJ* put it, that Maybury was ‘a bungler’.^110^ During an ‘unskilled’ surgery, Maybury violently dislocated Percy Powell’s shoulder.^111^ In their coverage of the court proceedings, the *BMJ* reported that Maybury had refused a second opinion, instead performing another operation. Despite it being unsuccessful, Maybury told Powell that ‘everything was going on well’. He also dissimulated to Powell’s daughter, an orthopaedic nurse, and refused to allow Powell to consult his own brother, another doctor. In desperation, Powell eventually consulted his brother and was admitted to the Royal Portsmouth Hospital. Although Burrows attempted a repair, Powell was ‘gravely handicapped’.^112^ Denying responsibility, Maybury contended that interference from Burrows and Powell’s relatives was to blame. According to Maybury, his ‘only fault, *if there was a fault*, was that he was too hopeful’.^113^ The jury was unconvinced.

Maybury may have resented interference but happily drew from the writings of specialists if they supported his case. He cited authorities, sometimes incorrectly, on the methods, suitability and safety of intravenous injections. For example, he claimed that neurologist Harry Campbell had ‘never known a case of clotting in a vein from NAB and he does not think that intravenous injections of NAB would in any way be harmful if the case was not one of syphilis’.^114^ Maybury claimed to have followed Campbell’s recommended .05 gm dosage for infants. But according to his own records, he injected Leslie with doses that ranged widely from an implausibly precise .01125 gm to .075 gm.^115^ He also acknowledged that Campbell ‘had no experience of longitudinal sinus injections’, preferring to inject children intramuscularly.^116^ Moreover, Campbell cautioned that intravenous injections of NAB were still experimental, ‘beset with many dangers’ and requiring ‘utmost caution’.^117^ When in 1919 Campbell and his collaborator, Charles Ballance, Surgeon to St Thomas’s Hospital, reported positive results, they were speaking about tabes dorsalis and general paralysis of the insane (GPI) among adults.^118^

Maybury’s counsel dismissed as ‘nonsense’ the claims that NAB injections into the longitudinal sinus were dangerous, adding that ‘experts recommended the operation as a highly safe and desirable one for introducing medicinal matter into the blood of an infant’.^119^ But venereologist James J. Abraham was an outlier when he testified as a defence witness that it was the best method.^120^ In reality, it was widely regarded as especially ‘dangerous and difficult’.^121^ According to one GP who appeared for the plaintiff, the procedure had ‘fallen into disrepute’ because the risk of brain injury was too great.^122^ Even many venereologists remained ambivalent.^123^ Among those who recommended NAB, few identified the longitudinal sinus as a suitable injection site.^124^ McDonagh cautioned against injections into the anterior fontanelle as ‘too heroic to find many adherents’.^125^ He used arsenobenzene reluctantly among infants and only when death was certain ‘unless something drastic is done’. When he did inject into the longitudinal sinus, he used .01 gm of a soluble salt in 5.0 c.cm of water.^126^ Unlike Maybury, he did not increase the dosage, seemingly at random.

According to Hart, the clinical quality of general practice was ‘simply ignored’ until 1952 when the Royal College of General Practitioners was established.^127^ But there had been decades of anxious rumblings about GPs’ capabilities. In 1914, the Royal Commission on Venereal Disease had raised concerns about the dangerous, unskilled use of new therapies. Appearing before the Commission, James Sequeira, an expert on dermatological syphilis, lamented that he was ‘constantly coming across men, even in good practices, who have not any idea of … things which are everyday knowledge to our third, fourth and fifth-year students. It is very difficult, of course, to keep these men up to date’.^128^ As Lane had put it when cautioning against the indiscriminate use of salvarsan, ‘even if the mortality after these injections is small relatively, and is due to want of discrimination in the selection of cases or to errors of technique, how are we going to obviate these errors in either judgement or in the method of administering the remedy’?^129^ When asked by the Commissioners how older doctors might be reached and taught to avoid such calamities, Sequeira believed that the only option was to wait for them to be replaced by the next generation.^130^

Maybury may, as Portsmouth’s MOH insisted, have possessed ‘the necessary qualifications’. But his reliance on the incorrectly cited (and possibly incorrectly understood) practices of other doctors suggests that his injections into the longitudinal sinus were based largely on book learning. Older doctors, especially those in general practice who trained before the development of serology and its incorporation into the medical curriculum, were far less equipped to employ these new technologies.^131^ Maybury qualified in 1879, decades before Wassermann testing, arsenobenzenes or the identification of the *spirochaeta pallida*.^132^ Although for many years he had divided his time between general practice and his work as Portsmouth’s police surgeon, neither role provided regular opportunities to systematically update his venereological knowledge or skill.^133^ Writing to the *BMJ* in 1918 about the shortcomings of venereology in general practice, an anonymous correspondent blamed the inadequacies of undergraduate medical training and the lack of accessible postgraduate courses.^134^ Even though a growing network of VD clinics offered GPs opportunities to augment their clinical knowledge, the necessary commitment was something that ‘a busy practitioner can ill afford in these strenuous days’.^135^ As Thomas Anwyl-Davies, the first director of the Whitechapel VD Clinic, put it pessimistically in 1935, ‘our knowledge of venereology has advanced so considerably of late years … that only the specialist devoting his whole time to its study can hope to keep abreast of the complex problems that arise in the treatment of venereal disease. To the busy GP, this more specialised knowledge is not possible’.^136^

## Confidentiality

Although Maybury sought permission to inject Leslie intravenously, he did not say what would be injected or what it was being injected for.^137^ In some respects, his actions conformed to professional standards of his time, shaped by an ethical framework that had yet to consolidate around patient safety, trust, good communication and informed consent.^138^ Abraham insisted that parents should be told if their child had syphilis. But in the next breath, he was adamant that Maybury had not been negligent in declining to inform Leslie’s parents.^139^ In the 1920s it was acceptable to communicate only the information necessary to cultivate trust and persuade patients of a recommended pathway. Even when Maybury refused to be candid with Marcus and Charlotte Shewry, they permitted Leslie’s treatment because, as Helyer testified, they trusted Maybury to ‘do their baby good’.^140^

Women were not infrequently cognisant of the terrible realities of VD. As one woman remarked bitterly to the venereologist Jonathan Hutchinson, her husband ‘denied having had it and made excuses’, but she ‘was not such a fool as to believe him’.^141^ Nonetheless, it was common for doctors to withhold diagnoses of VD from wives and mothers on the grounds that doing otherwise might cause domestic strife.^142^ According to Maybury, he did not test Leslie’s parents because ‘a positive reaction … would possibly break up the home’.^143^ Such attitudes had shaped medical decision making for decades.^144^

But even when concealment from women was commonplace, it was unusual for Maybury to withhold his diagnosis from Leslie’s father—a decision that Maybury justified through the curious invocation of ‘professional etiquette’.^145^ That Maybury’s solicitor, Oswald Hempson, asked Eberlie to confirm the rightness of Maybury’s decision suggests that he anticipated it becoming a point of contention in court: ‘Dr Maybury in his discretion did not think it necessary or indeed advisable to inform the parents of this diagnosis and we shall be glad to know that you agree that this was justifiable and indeed a wise admission’.^146^ It did indeed become an issue, so much so that Hewart had to remind the jury that it was not their job ‘to decide upon medical ethics’.^147^

It was widely accepted that something as serious as syphilis would be disclosed to husbands and fathers. As the people usually responsible for paying the doctor’s fees, they theoretically had leverage in the clinical relationship and might expect to be taken into the doctor’s confidence. Their cooperation was also considered necessary to facilitate treatment.^148^ Regarded as ‘the hereditary disease *par excellence*’, syphilis presented a unique threat to the health of the nation, making such disclosures all the more imperative.^149^ But asking a fee-paying patient if he had ever had VD was undoubtedly awkward and probably bad for business. He might construe the questioning as impertinent or a slight on his character and go elsewhere. According to Maybury, it was ‘almost impossible in the social married life of people to enquire about this disease’, suggesting that he felt particular embarrassment at the prospect. He went on the insist that, fortunately, there was ‘no need when the clinical signs are diagnostic’.^150^ But even if there were diagnostic indicators of congenital syphilis, it was expected that doctors would make every effort to establish the health of both parents—a necessary undertaking requiring a tactful, but nonetheless frank, exchange.^151^

For Leslie to be born with aggressive congenital syphilis, Charlotte Shewry would probably have been symptomatic. Yet Maybury, who attended her during her pregnancy, had observed no signs of infection.^152^ Indeed, she subsequently had two healthy children. Fear of syphilis’s hereditary implications meant that pregnant women suspected of having it were often given prophylactic treatment.^153^ As one doctor put it in 1920, ‘antenatal treatment is the only rational method of treating congenital syphilis’.^154^

Why then did Maybury conceal his diagnosis if it meant withholding treatment from Leslie’s parents, potentially risking their health and the health of future children? Why did he deal with Shewry in the same paternalistic way that many fellow doctors dealt with women and children? Maybury was content not to look beyond Leslie’s supposedly ‘diagnostic’ symptoms because doing so would have necessitated an embarrassing conversation. But there was also another explanation. The early twentieth century saw what Hart describes as a ‘virtual denial of any active and intelligent role for patients’, especially lower-middle-class and working-class patients.^155^ Maybury, who maintained a hierarchical relationship with his patients and expected them to submit unquestioningly to his authority, was no exception.

Having cited confidentiality as the reason for concealing Leslie’s diagnosis, Maybury subsequently showed complete indifference to its necessity. He spent many hours hunting down Leslie’s medical records for the years after he ceased to be his doctor, hoping they might be ‘a very fruitful vine’ for his defence. Maybury also capitalised on the fact that Hempson’s brother was Medical Officer at the hospital where Shewry was initially receiving treatment, unscrupulously soliciting detailed reports of Shewry’s cancer and, by extension, his ability to instruct his counsel. He then freely shared this information with Eberlie, while having only recently and apologetically declined to share a report that Macpherson had asked Maybury to keep confidential.^156^ In matters of confidentiality, Maybury showed his fellow doctors a courtesy that he did not extend to patients. He cynically exploited the notion of confidentiality without consideration for Leslie, Charlotte or Marcus Shewry, the latter of whom he described contemptuously as ‘not very smart for an inspector’.^157^

## Networks

Despite or possibly because of its inconclusiveness, Shewry v. Maybury attracted much attention in the medical press, including numerous pieces of correspondence from readers anxious about the case’s implications for their own livelihoods.^158^ Laid bare to legal and public scrutiny, such cases embodied what Claire Brock aptly describes as ‘the contemporary nightmares experienced by the profession itself’.^159^

Such responses were neither unexpected nor unusual. As in previous negligence cases, reporting favoured the defendant as a fellow doctor.^160^ The *BMJ* cast doubt on the Shewrys’ medical witnesses while describing Maybury’s as ‘gentlemen of high position in the profession’.^161^ The *Lancet* was equally unsubtle with claims like ‘medical witnesses for the defence gave evidence which one would suppose no jury could reject’.^162^ That the jury did reject much of it was, according to the journals, a miscarriage of justice. One indignant correspondent insisted that, ‘before a doctor can be found to have been negligent, he must have fallen below the ordinary standard of his profession’, implying that Maybury had not done so.^163^ But before the 1957 Bolam decision, doctors’ professional actions were not judged legally against a professional standard, but according to what an ordinary or ‘reasonable’ layman might have done under the same circumstances.^164^

Most correspondents were outraged that a doctor’s actions had been scrutinised so publicly and that 12 laymen had been tasked with ‘the seeming miracle of deciding what was the proper treatment’ for an infant eight years earlier.^165^ It was considered unacceptable that people without medical qualifications had been authorised ‘to pass judgement upon the propriety of a medical practitioner’s diagnosis and treatment’.^166^ In finding Maybury negligent, the jury had, according to various correspondents, clearly allowed emotions to cloud their judgement.

The plaintiff’s case was opened to the jury as that of a poor little innocent child blasted at its birth by an incompetent doctor needlessly performing a most hazardous operation. Every mother who read the opening day’s proceedings must have thought Dr Maybury a fiend incarnate.^167^

Another *Lancet* correspondent, who signed themselves only as ‘Lex’ (but whose partiality and familiarity suggested an intimate association with the defence) offered an equally impassioned rebuke: if the jury ‘had listened attentively … not forgetting they had sworn to find a verdict upon evidence given, they would now be happier and wiser, sentiment and prejudice having no place in the finding of their verdict’.^168^ Some even called for the jury system to be overhauled in negligence cases, proposing instead a system of medical assessors who would filter technical evidence and present their conclusions to the jury.^169^ A. Knyvett Gordon, who had appeared as a defence witness, wrote that ‘it is the details that worry members of a jury and are apt to lead to confusion, which often results in a prejudiced opinion in a certain type of mind’.^170^

But prejudicial opinions were also influencing minds beyond those of the jury. Maybury’s counsel objected to the Shewrys’ medical witnesses, who ‘came on the scene some years after’ Leslie’s treatment, allegedly rendering their opinions unsound.^171^ But Maybury had also called witnesses to defend clinical actions about which they had no first-hand knowledge.^172^ His primary concern was finding ‘*any* men of authority who would help if required’.^173^ He believed that he had found such a man in Gordon, who was useful because he was experienced in court proceedings and could assertively ‘hold his own against any man at the Bar’.^174^ Some defence witnesses also appear to have based their opinions largely on their professional or personal relationships with Maybury. Macpherson, for example, insisted that:

I have heard from Dr Maybury … that this infant was suffering from congenital syphilis and in my opinion, this was a perfectly justifiable diagnosis despite the fact that the Wassermann reaction test was negative. Dr Maybury has also described to me in detail the treatment given by him … The technique was perfect and beyond question … I have known Dr Maybury and his work for some time and am of the opinion that he is a skilled practitioner and well capable of carrying out the treatment in this case which seems to me to have been appropriate and in every way right and proper.^175^

But how could Macpherson be so confident if he had neither seen Leslie as an infant nor witnessed the injections? Apparently, Maybury had simply shown him a syringe similar to the one used on Leslie, prompting Macpherson to conclude that, ‘if such a needle and syringe were used … in my opinion they would not cause or conduce the present condition of Leslie Shewry’.^176^

Others from whom Maybury solicited support were less obliging—a fact he resented.

I have obtained tons of correspondence from most of the experts in London. Some agreed on one point and some on another but no one expert on all points. Everybody was sympathetic but did not want to go to court to be heckled and badgered. I often think of what I have done for men in difficulties but then, I suppose I love a fight and have the inclination to help others.^177^

Certainly, doctors were and remain reluctant to become embroiled in legal proceedings.^178^ But in this instance, as Maybury acknowledged to Eberlie, ‘most experts on syphilis are emphatic that a case is not syphilitic unless there is a positive Wassermann, whatever the clinical signs’.^179^

In contrast to his polite requests of fellow doctors, Maybury sought to intimidate the nurse who had attended Leslie’s birth. He hired a private detective to question Martha Sturgess, but was frustrated to find her adamant that Leslie had been born healthy.^180^ Under oath, she testified that Maybury himself had declared Leslie to be ‘a fine, healthy boy’ and that it was only ‘after the injection the baby rapidly went back in health and it had bad fits and convulsions’.^181^

In his efforts to control the narrative, Maybury micromanaged witness statements. ‘To save you time’, he explained to Macpherson, ‘I have tabulated some points and if you will be kind enough to fill them in you need not keep a copy as I will send you a copy of the points and your replies’.^182^ Such interventions were most evident, however, in his dealings with Eberlie. Throughout their correspondence Maybury included pointed reminders of Eberlie’s role in Leslie’s diagnosis and treatment. He even dictated what Eberlie should include in his witness statement.

Had we not diagnosed syphilis, we should not have treated for syphilis. We took every care as regards sterility and mode of operation … You will remember in all these cases we used an Allen and Hanbury glass 10cc syringe with a short needle and a very short bevel and we always drew blood into the syringe after the longitudinal sinus had been entered. With the technique of the operation, it would be absolutely impossible to puncture the longitudinal sinus and inject some of the NAB solution into the subarachnoid space or into the brain … In your proof, I should like you to mention that you served in the Royal Navy three years and that you assisted me in my practice … Mention you had experience of syphilis and NAB in the Navy and the experience you had with me with adults and infants, that the technique and injections were perfect and that we never had any ill effects from injections of NAB. If you can remember Leslie Shewry then please state that the injections were skilfully and not negligently performed and that they did not cause or conduce to the present state of Leslie Shewry.^183^

In a subsequent letter, Maybury thanked Eberlie for his statement, which he had taken the liberty of editing before it was submitted to the solicitors.^184^ And as the court date approached, Maybury began stage-managing Eberlie’s testimony.^185^ Not only did he compile a list of questions that he suspected might be put to Eberlie, but he also considerately supplied him with answers to those questions.^186^

## Conclusions

In summing up, Hewart reminded the jury that ‘they were engaged in trying a case of exceptional gravity’.^187^ Although agreeing that Maybury had been negligent, both in his diagnosis and general treatment, they could not agree on the principal question of whether Leslie’s condition resulted from that treatment.^188^ On 26 July, Hewart ruled that he could not enter a judgement for either party.^189^

But by that point, Leslie was dead. He had died on 13 July. Macpherson, who certified his death, registered four causes: bronchopneumonia, exhaustion, epilepsy and Little’s Disease.^190^ Shewry died only a few weeks later on 3 August at his home in Portsmouth.^191^ He had given evidence from hospital and, although discharged by the time the case was heard, was too sick to attend court.^192^ Aware that he might be dying, he had brought the case in the hope that, through damages, Leslie’s ‘future might be made as happy as possible’.^193^ But far from receiving damages, the Shewrys were almost ruined.

Maybury was far more financially secure. At the time of the 1921 census, his household included four servants.^194^ By contrast, the Shewrys lived in a modest terraced house in a far less affluent area.^195^ After Shewry’s death, Charlotte eventually remarried and she and her surviving children went to live with her new husband, an omnibus conductor.^196^ When Maybury died in 1937, aged 82, his estate was worth £26,632.^197^ His greater wealth and social capital, combined with his ability to exploit legal processes and a network of eminent medical colleagues, stacked the cards in his favour. The chances that the Shewrys might successfully challenge his medical authority were always going to be slim. Yet as the court case progressed, Maybury nonetheless felt a strain on his nerves and finances. Having not been a member of the Medical Defence Union when the alleged negligence occurred, he was obliged to fund his own defence.^198^

It is likely that Maybury had not intended to harm Leslie. If we believe his account, even his decision to conceal Leslie’s diagnosis was to prevent distress. Nonetheless, harm was done. While we cannot determine whether his actions caused Leslie’s lifelong disabilities, they set in motion a series of events that caused harm. His subsequent efforts to control and reshape the clinical narrative were calculated to protect his reputation and achieve a favourable outcome for himself, regardless of any additional harm to the Shewrys. As a police surgeon called on regularly to give evidence, he understood court and legal processes and was able to work them to his advantage. And in this, he was aided by a system that allowed him to choreograph his defence. He communicated directly with witnesses, influenced their recollections and even amended their witness statements.

On one level, the case hinged on the word of a doctor against that of his patient, and on the contentious claims of medical witnesses who simply could not agree; this was a family’s search for redress in the face of a medical profession closing ranks. But on another level, it was a clash between medicine’s rapid technologisation and the inability and unwillingness of some doctors to keep pace. Technologies like NAB and the Wassermann reaction promised to radically reshape venereology. But without adequate regulatory controls, they also created an environment for clinical practices that were inherently risky by virtue of the egotism, profiteering and inexperience of the doctors deploying them. GPs like Maybury were able to exploit their professional standing and status to justify working beyond their capabilities and expertise. Their perceptions of their own expertise could be at odds with the needs of patients and emerging forms of medical knowledge and practice. And in Leslie’s case, the consequences were grievous. Maybury’s arrogance, combined with the medical profession’s instinct to protect its own, created a situation in which he was able to negligently treat and possibly harm a newborn without serious professional consequences.

## Footnotes

1 Wellcome Collection, Papers of Felix Eberlie Regarding the Case of Shewry v. Maybury 1929, Writ issued the 23rd day of June 1928: Statement of Claim, MS.8792/1. [Henceforth Shewry v. Maybury].

2 Ibid.

3 Ibid.

4 This article concerns three people with the same surname. While it might be expedient to use just their first names, doing so would have the effect of leaning into the hierarchy that existed between Maybury, a medical professional, and the Shewrys who lacked the same educational and social advantages. To remove possible confusion, but also to avoid the replication of these hierarchies, Marcus will be referred to as Shewry, Charlotte will be referred to as Charlotte Shewry and Leslie will be referred to by just his first name.

5 Ibid.

6 Ibid.

7 The value and limitations of microhistory for understanding historical phenomena have been extensively debated. For example, Peter Jones, ‘Looking Through a Different Lens: Microhistory and the Workhouse Experience in Late Nineteenth-Century London’, *Journal of Social History* 2022, 55, 925–47; Sigurdur Gylfi Magnússon, ‘New and Old Theoretical Issues: Criticisms of microhistory’, in Sigurdur Gylfi Magnússon and István M. Szijártó (eds), *What Is Microhistory? Theory and Practice* (London: Taylor & Francis, 2013), 119–33; John Brewer, ‘Microhistory and the Histories of Everyday Life’, *Cultural and Social History* 2010, 7, 87–109.

8 For example, Jonathan Reinarz and Rebecca Wynter (eds), *Complaints, Controversies and Grievances in Medicine: Historical and Social Science Perspectives* (London: Routledge, 2015); Louise Hide and Joanna Bourke, ‘Cultures of Harm in Institutions of Care’, Special Issue of *Social History of Medicine* 2018, 31; Sally Sheard, ‘Can We Never learn? Abuse, Complaints and Inquiries in the NHS’, History & Policy Opinion Articles (26 February 2015). www.historyandpolicy.org/opinion-articles/articles/why-we-never-learn-abuse-complaints-and-inquiries-in-the-nhs. Accessed October 2023.

9 Christopher Sirrs, ‘The Moment of Patient Safety: Iatrogenic Injury, Clinical Error and Cultures of Healthcare in the NHS’, *Social History of Medicine,* 2024, 37, 93–115; Hide and Bourke, ‘Cultures of Harm’, 684.

10 Portsmouth History Centre, *The Evening News*, ‘Claim Against a Southsea Doctor: Police Inspector’s Child’ (12 June 1929).

11 Daniel Grey, ‘Murder, Mental Illness, and the Question of Nursing “Character” in Early Twentieth Century England’, *History Workshop Journal*, 2015, 80, 183–200; Kim Price, *Medical Negligence in Victorian Britain: The Crisis of Care under the English Poor Law, 1834–1900* (London: Bloomsbury, 2015); Paul Carter and Steve King, ‘The patient’s view as history from below: evidence from the Victorian poor, 1834–71’, Anne Hanley and Jessica Meyer (eds), *Patient Voices in Britain, 1840–1948* (Manchester: Manchester University Press, 2021), 154–80. For general practice, see Alannah Tomkins, *Medical Misadventure in an Age of Professionalisation* (Manchester: Manchester University Press), 117–98.

12 Following Bolam, a doctor could no longer be found guilty of negligence if their actions were deemed to have been in accordance with the accepted or ‘reasonable’ standards of their day, meaning that even a minority medical opinion might constitute a viable defence. See Margaret Brazier and José Miola, ‘Bye Bye Bolam: A Medical Litigation Revolution?’, *Medical Law Review,* 2000, 8, 85–114; Price, *Medical Negligence in Victorian Britain*, 2–6.

13 The iceberg metaphor is used often in discussion of healthcare, including complaints about care standards. For example, Rudolf Klein, *Complaints against Doctors: A Study in Professional Accountability* (London: Charles Knight, 1973); Kim Price, ‘The Shape of the Iceberg: Doctors and Neglect under the New Poor Law, 1871–1900’, Reinarz and Wynter, *Complaints*, 129–46.

14 Challenges that are examined meticulously in Susan C. Lawrence, *Privacy and the Past: Research, Law, Archives, Ethics* (New Jersey: Rutgers University Press, 2016). See also Jessica Meyer and Alexia Moncrieff, ‘Family not to be informed? The ethical use of historical medical documentation’, Anne Hanley and Jessica Meyer (eds), *Patient Voices in Britain, 1840–1948* (Manchester: Manchester University Press, 2021), 61–87.

15 E.P. Thompson, *The Making of the English Working Class* (London: Penguin, reprint 2013), preface.

16 For more on the methodological and ethical challenges of this, see Julia Laite, ‘The Emmet’s Inch: Small History in a Digital Age’, *Journal of Social History*, 2020, 53, 963–89.

17 Richard A. McKay, ‘Becoming an Ethical Historian’, Courtney E. Thompson and Kylie M. Smith (eds), *Do Less Harm: Ethics for Health Historians* (Baltimore: Johns Hopkins University Press, 2025).

18 Katie Sloan et al., ‘Not “Just My Problem to Handle”: Emerging Themes on Secondary Trauma and Archivists’, *Journal of Contemporary Archival Studies*, 2019, 6.

19 For more on this, see Sarah Fox, ‘Archival Intimacies: Empathy and Historical Practice in 2023’, *Transactions of the Royal Historical Society*, 2023, 241–65.

20 For example, Charles E. Rosenberg, ‘The Tyranny of Diagnosis: Specific Entities and Individual Experience’, *The Milbank Quarterly*, 2002, 80, 237–60; Andrew Cunningham, ‘Identifying Disease in the Past: Cutting the Gordian Knot’, *Asclepio*, 2002, 54, 13–34; Tracey Loughran, ‘Shell Shock, Trauma, and the First World War: The Making of a Diagnosis and Its Histories’, *Journal of the History of Medicine and Allied Sciences*, 2012, 67, 94–119; Osamu Muramoto, ‘Retrospective Diagnosis of a Famous Historical Figure: Ontological, Epistemic, and Ethical Considerations’, *Philosophy, Ethics and Humanities in Medicine*, 2014, 9.

21 Chris Millard, ‘Concepts, Diagnosis and the History of Medicine: Historicising Ian Hacking and Munchausen Syndrome’, *Social History of Medicine*, 2017, 30, 586.

22 Rosenberg, ‘The Tyranny of Diagnosis’, 252–53.

23 Rosenberg, ‘The Tyranny of Diagnosis’, 241, 250. See also Charles E. Rosenberg, ‘Disease in History: Frames and Framers’, *The Milbank Quarterly*, 1989, 67, 10.

24 MS.8792/1.

25 Shewry v. Maybury, Letter from Maybury to Eberlie on 25 July 1928, MS.8792/3.

26 Anon., ‘Charge of Professional Negligence: Shewry v. Maybury’, *BMJ* (29 June 1929), 1185.

27 Thomas Ballard, *An Enquiry into the Value of the Signs and Symptoms Regarded as Diagnostic of Congenital Syphilis in the Infant* (London: J. & A. Churchill, 1874), 7–8. See also J.A. Coutts, *Some Aspects of Infantile Syphilis: Being the Hunterian Lectures Delivered at the Royal College of Surgeons in 1896* (London: Rivington, Percival & Co, 1897), 51; Pernet George, *Differential Diagnosis of Syphilitic and Non-Syphilitic Affections of the Skin, including Tropical Diseases: A Survey for Medical Practitioners and Students* (London: Adlard, 1904), 164.

28 George, *Differential Diagnosis*, 171. See also Coutts, *Some Aspects of Infantile Syphilis*, 71–72; Ernest R.T. Clarkson (ed.), *The Venereal Clinic: The Diagnosis, Treatment and Prevention of Syphilis and Gonorrhoea. A Handbook of Venereal Disease in Relation to the Individual and the Community* (London: J. Bale, Sons & Danielsson, 1922), 42–43; Hubert Armstrong, ‘On Some Clinical Manifestations of Congenital Syphilis’, *BMJ,* 1914, 958–61; L.W. Harrison, *The Diagnosis and Treatment of Venereal Diseases in General* Practice (London: Hodder & Stoughton, 1919), 216, 226; J.E.R. McDonagh, *Venereal Diseases: Their Clinical Aspect and Treatment* (London: William Heinemann, 1920), 181–84.

29 Coutts, *Some Aspects of Infantile Syphilis*, 67.

30 Armstrong, ‘On Some Clinical Manifestations’, 959.

31 Ballard, *An Enquiry*, 14.

32 Ibid.

33 McDonagh, *Venereal Diseases*, 183.

34 George, *Differential Diagnosis*, 166; See also McDonagh, *Venereal Diseases*, 181.

35 Coutts, *Some Aspects of Infantile Syphilis*, 45. See also Armstrong, ‘On Some Clinical Manifestations’, 960.

36 Jonathan Hutchinson, a leading authority on venereal diseases, defined three characteristic indicators of congenital syphilis in children. See Jonathan Hutchinson, *Syphilis* (London: Cassell, 1887), 431–40.

37 Anon., ‘Charge of Professional Negligence’, 1185.

38 For more on the Wassermann test and its use in clinical practice, see Anne Hanley, *Medicine, Knowledge and Venereal Diseases in England, 1886–1916* (London: Palgrave, 2017), 126–34, 147–73.

39 Medical Research Committee, Reports of the Special Committee upon the Standardisation of Pathological Methods. No.1: The Wassermann Reaction Interim Report (March 1918); Pauline M.H. Mazumdar, ‘“In the Silence of the Laboratory”: The League of Nations Standardises Syphilis Tests’, *Social History of Medicine*, 2003, 16, 437–59.

40 Paul Fildes and Roderick J.G. Parnell, *An Analysis of the Results of Wassermann Reactions in 1435 Cases of Syphilis or Suspected Syphilis: Special Report of the Medical Research Committee* (HMSO, 1919); Anon., ‘The Significance of the Wassermann Reaction’, *Lancet* (10 May 1919), 807.

41 Medical Research Committee, Accuracy of the Wassermann Tests applied before and after death, estimated by Necropsy: 1. The Wassermann Test applied before Death (January 1920), 3–4.

42 Ibid, 89.

43 Shewry v. Maybury, Letter from Maybury to Eberlie on 15 February 1929, MS.8792/10.

44 MS.8792/3.

45 Anon., ‘Charge of Professional Negligence’, 1186.

46 Shewry v. Maybury, Letter from Maybury to Macpherson on 15 November 1928 and Macpherson’s proofs, MS.8792/7.

47 Ibid.

48 Ibid.

49 Shewry v. Maybury, Letter from Maybury to Eberlie on 30 November 1928, MS.8792/9. Emphasis added.

50 C.H. Browning and E.L. Kennaway, ‘Wassermann Tests: The Occurrence of Variations in the Result of Successive Tests; The Clinical Application of the Data’, *Lancet*, 1919, 785–86; Royal Commission on Venereal Diseases [henceforth RCVD], Appendix to First Report of the Commissioners, Minutes of Evidence PP 1913–16 Cd 7475, qq. 11384–86; RCVD, Appendix to Final Report of the Commissioners, Minutes of Evidence PP 1913–16 Cd 8190, qq. 13645–46.

51 Rosenberg, ‘The Tyranny of Diagnosis’, 247.

52 C.F. Marshall and E.G. Ffrench, ‘The Early Diagnosis of Syphilis’, *BMJ*, 1920, 688.

53 Hugh Wansey Bayly, ‘The Value of the Wassermann Reaction’, *BMJ,* 1921, 686. Lock Hospitals were specifically for the treatment of VD among the working classes. See Frederick Lowndes, *Lock Hospitals and Lock Wards in General Hospitals* (London: J&A Churchill, 1882).

54 Hutchinson first described in 1879 the idea that syphilis ‘imitated’ other diseases. See Jonathan Hutchinson, ‘An Address on Syphilis as an Imitator’, *BMJ*, 1879, 499–501; ‘An Address on Syphilis as an Imitator (Concluded)’, *BMJ,* 1879, 541–42.

55 James Ernest Lane, ‘Discussion on Recent Developments in the Recognition and Treatment of Syphilis: Held in Conjunction with the Section on Dermatology’, *BMJ,* 1911, 673.

56 Shewry v. Maybury, Eberlie’s statement, MS.8792/5.

57 J.E.R. McDonagh, ‘Cases of Syphilis Illustrating the Importance of the Wassermann Reaction’, *Journal of the Royal Society of Medicine,* 1910, 3, 91.

58 McDonagh expressed his scepticism and unease about Wassermann testing on many occasions and in multiple forums. See, for example: RCVD, Appendix to Final Report, qq. 15919–26, 16027–29, 16061–64, 16153–59; J.E.R. McDonagh, ‘The Action of Salvarsan and Neosalvarsan on the Wassermann Reaction’, *BMJ*, 1912, 1287–89; ‘The Rationale or Modus Operandi of the Wassermann Reaction and Abderhalden’s Test’, *QJM: An International Journal of Medicine,* 1915, 8, 129–55; ‘The Rationale of the Wassermann Reaction: An Abridged Account of a Paper Read Before the Dermatological Section of the Royal Society of Medicine on October 20th 1921’, *The Lancet*, 1921, 1319–22.

59 MS.8792/10.

60 Rosenberg, ‘The Tyranny of Diagnosis, 240.

61 Shewry v. Maybury, Letter from Maybury to Eberlie on 31 July 1928, MS.8792/4.

62 MS.8792/5.

63 MS.8792/1; MS.8792/5.

64 Shewry v. Maybury, Letter from Hempsons Solicitors to Dr Eberlie, MS.8792/6.

65 Anon., ‘Charge of Professional Negligence’, 1186. See also W.B. Cosens, ‘Shewry v. Maybury: To the Editor of the Lancet’, *Lancet*, 1929, 147.

66 Anon., ‘Charge of Professional Negligence’, 1186.

67 A Correspondent, ‘Note-Taking in General Practice’, *BMJ*, 1921, 430–31.

68 MS.8792/3.

69 MS.8792/1.

70 ‘Claim Against a Southsea Doctor’.

71 MS.8792/1.

72 Ibid.; MS.8792/3.

73 ‘Claim Against a Southsea Doctor’.

74 See, for example, Lane, ‘Discussion on Recent Developments’, 675; Clarkson (ed.), *The Venereal Clinic*, 60; McDonagh, *Venereal Diseases*, 235–36.

75 Julian Tudor Hart, ‘Going to the Doctor’, Roger Cooter and John Pickstone (eds), *Medicine in the Twentieth Century* (London: Routledge, 2000), 544.

76 For more on the development of these drugs, see J.E. Ross and S.M. Tomkins, ‘The British Reception of Salvarsan’, *Journal of the History of Medicine and Allied Sciences*, 1997, 52, 398–423; Hanley, *Medicine, Knowledge and Venereal Disease*, 117–26.

77 L.W. Harrison, C.F. White and C.H. Mills, ‘The Intramuscular or Subcutaneous Injection of Neosalvarsan’, *BMJ*, 1917, 569.

78 The clinical apparatus and procedures for administering arsenicals were so complex that the attending doctor required specially trained assistants, who would be able to help arrange the treatment space, prepare the drugs for injection and prevent their oxidation, assist in their administration and support post-operative patient care. See, for example, George Stopford-Taylor and Robert W. MacKenna, *The Salvarsan Treatment of Syphilis in Private Practice, with some Account of the Modern Methods of Diagnosis* (London: William Heinemann, 1914), 37–47; Hugh W. Bayly, ‘The Control of Venereal Diseases: The Venereal Clinic, Its Organisation and Administration’, *Lancet*, 1917, 690–92; Alexander Fleming and L. Colebrook, ‘On the Use of Salvarsan in the Treatment of Syphilis’, *Lancet*, 1911, 1631–34; Harrison, *The Diagnosis and Treatment of Venereal Diseases in General Practice*, 362–66.

79 James McIntosh and Paul Fildes, *Syphilis from the Modern Standpoint* (London: Edward Arnold, 1911), 187.

80 Lane, ‘Discussion on Recent Developments’, 678.

81 Anon., ‘Annual Meeting of the British Medical Association. Section of Venereal Diseases: Discussion on Venereal Diseases in Women and Children’, *BMJ,* 1920, 201.

82 Harrison, *The Diagnosis and Treatment of Venereal Diseases*, 375; Clarkson (ed.), *The Venereal Clinic*, 58–61; McDonagh, *Venereal Diseases*, 228–46.

83 McDonagh, *Venereal Diseases*, 209; Lane, ‘Discussion on Recent Developments’, 675.

84 William Hardman, ‘“Ehrlich-Hata” or “606”’, *BMJ*, 1911, 54–55; J.E. Ross and S.M. Tomkins, ‘The British Reception of Salvarsan’, *Journal of the History of Medicine and Allied Sciences*, 1997, 52, 401–10; Hanley, *Medicine, Knowledge and Venereal Disease*, 117–25, 147–87.

85 Tudor Hart, ‘Going to the Doctor’, 551.

86 Anon., ‘Charge of Professional Negligence’, 1186.

87 Ibid, 1185.

88 Ibid.

89 Ibid; MS.8792/1; Shewry v. Maybury, Letter to Eberlie on 16 June 1929, including John Lane’s Proof, MS.8792/15.

90 Portsmouth History Centre, *The Evening News*, ‘Police Officer’s Child: The Claim Against a Southsea Doctor’ (14 June 1929); MS.8792/1.

91 For more on the establishment and organisation of the VD Service, see Roger Davidson, *Dangerous Liaisons: A Social History of Venereal Disease in Twentieth-Century Scotland* (Amsterdam: Rodopi, 2000); David Evans, ‘Tackling the ‘Hideous Scourge’: The Creation of the Venereal Disease Treatment Centres in Early Twentieth-Century Britain’, *Social History of Medicine,* 1992, 5, 413–433; Anne Hanley, ‘Sex Prejudice’ and Professional Identity: Women Doctors and their Patients in Britain’s Interwar VD Service’, *Journal of Social History,* 2020, 54, 569–598.

92 Anne Digby, *The Evolution of British General Practice, 1850–1948* (Oxford: Oxford University Press, 2008), 94.

93 Anon., ‘General Practitioners and Venereal Disease’, *BMJ*, 1918, 119.

94 F.J.G. Jefferiss, ‘The Return of the Venereal Diseases’, *BMJ*, 1962, 1751.

95 Anon., ‘General Practitioners and Venereal Disease’, 119.

96 ‘Police Officer’s Child’.

97 Hanley, *Medicine, Knowledge and Venereal Diseases*, 71.

98 Anon., ‘Charge of Professional Negligence’, 1186.

99 Portsmouth History Centre, *The Evening News*, ‘Action Against Southsea Doctor: The Concluding Stages’, (19 June 1929).

100 Hanley, *Medicine, Knowledge and Venereal Diseases*, 120–21, 169.

101 Wellcome Collection, Burroughs Wellcome & Co, *Price List of Fine Products* (1930) 150, 152.

102 Anon., ‘Charge of Professional Negligence’, 1186.

103 Anon., ‘Charge of Professional Negligence’, 1186. In 1920, 5 guineas were fifteen days’ income for a skilled tradesman. See The National Archives Currency Converter: www.nationalarchives.gov.uk/currency-converter. Accessed July 2023.

104 Tudor Hart, ‘Going to the Doctor’, 545.

105 Shewry v. Maybury, Letter to Eberlie on 13 May 1929 and Maybury’s comments on Marcus Shewry’s evidence, MS.8792/13; Portsmouth History Centre, *The Evening News*, ‘Southsea Doctor Sued: Action by Boy of Nine. Son of Portsmouth Police Inspector’ (11 June 1929).

106 Anon., ‘Appendix: Report by Medical Secretary on Investigation into the Operation of Maternity and Child Welfare Centres and School Clinics in Certain Areas’, *Supplement to the BMJ* (3 November 1928), 192.

107 House of Commons Parliamentary Papers, 1917–18. A bill intituled an act to prevent the treatment of venereal disease otherwise than by duly qualified medical practitioners, and to control the supply of remedies therefor; and for other matters connected therewith. Brought from the Lords 15 March 1917.

108 Anon., ‘Lay Persons and Birth Control’, *Westminster Gazette* (17 July 1923).

109 Anon., ‘A Loftier Race. Dr Marie Stopes at Portsmouth’, *The Evening News* (16 July 1923).

110 Anon., ‘An Action for Negligence: Powell v. Maybury’, *BMJ*, 1925, 43.

111 Ibid.

112 Ibid.

113 Ibid. Emphasis added.

114 MS.8792/3.

115 MS.8792/1.

116 MS.8792/3.

117 Campbell, ‘The Treatment of Parenchymatous Syphilis’, 1531; Harry Campbell, ‘Intrathecal Treatment with Salvarsanised Serum’, *Lancet*, 1914, 1648; Harry Campbell, ‘The Treatment of Parenchymatous Syphilis by Intracranial Medication’, *Lancet,* 1914, 1529–31; Charles A. Ballance, ‘A Method by which Remedial Agents may be Mingles with the Cerebral Cerebrospinal Fluid’, *Lancet*, 1914, 1525–29.

118 Harry Campbell and Charles A. Ballance, ‘The Treatment of Venereal Paralysis of the Insane by the Introduction of Salvarsanised Serum into the Lateral Ventricle and a Suggestion for the Arrest of Tabetic Optic Atrophy by the Same Means’, *Lancet*, 1919, 608–09.

119 Portsmouth History Centre, *The Evening News*, ‘Action against Southsea Doctor: A Baby’s Skull in Court’ (13 June 1929).

120 Anon., ‘Charge of Negligence against a Practitioner’, *Lancet* (29 June 1929), 1376. See also Anon., ‘Charge of Professional Negligence’, 1186–87.

121 Alan Moncrieff, ‘Longitudinal Sinus Wedge’, *Lancet* (5 November 1927), 976.

122 Anon., ‘Charge of Professional Negligence’, 1185. See also ‘Action against Southsea Doctor’.

123 See, for example, Clarkson (ed.), *The Venereal Clinic*, 70; John Adams, ‘Treatment of Antenatal and Postnatal Syphilis’, *BMJ,* 1918, 541–42.

124 For example, Leonard Findlay, ‘The Treatment of Syphilis in Infancy and Childhood. Annual Meeting of the British Medical Association. Section of Venereal Diseases: Discussion on Venereal Diseases in Women and Children’, *BMJ,* 1920, 198–99. See also Clarkson (ed.), *The Venereal Clinic*, 70.

125 McDonagh, *Venereal Diseases*, 254–55.

126 Ibid, 256.

127 Tudor Hart, ‘Going to the Doctor’, 545.

128 RCVD, Appendix to Final Report, q. 14411.

129 Lane, ‘Discussion on Recent Developments’, 675.

130 RCVD, Appendix to Final Report, qq. 14414–15.

131 Anne Hanley has written at length about the generational divides that affected the acquisition of new venereological knowledge and skill. See Hanley, *Medicine, Knowledge and Venereal Diseases*, 25–106.

132 Anon., ‘Medical News’, *BMJ,* 1876, 494; Anon., ‘Medical News’, *BMJ,* 1877, 746; Anon., ‘Medical News’, *BMJ,* 1878, 116. Now known as the treponema pallidum, the *spirochaeta pallida* was identified in 1905 as the causative microorganism of syphilis.

133 Although police surgeons had carried out enforced genital examinations under the Contagious Diseases Acts and were being called with increasing frequency as expert witnesses in cases where venereal disease was communicated through sexual assault, they were not experts on venereal disease. See Victoria Bates ‘“So Far as I Can Define without a Microscopical Examination”. Venereal Disease Diagnosis in English Courts, 1850–1914’, *Social History of Medicine*, 2013, 26, 38–55; Joanna Bourke, ‘Police Surgeons and Victims of Rape: Cultures of Harm and Care’, *Social History of Medicine*, 2018), 31, 711–31.

134 Anon., ‘General Practitioners and Venereal Disease’, 119; Hanley, *Medicine, Knowledge and Venereal Diseases*, 25–62.

135 John Alcindor, ‘General Practitioners and Venereal Disease’, *BMJ*, 1918, 176. See also Clarkson (ed.), *The Venereal Clinic*, 351.

136 Thomas Anwyl-Davies, *The Treatment of Venereal Disease in General Practice* (London: John Bale, Sons & Danielsson, 1935), v–vi.

137 MS.8792/1.

138 See, for example, Anne Hanley, ‘Syphilization and Its Discontents: Experimental Inoculation against Syphilis at the London Lock Hospital’, *Bulletin of the History of Medicine, *2017, 91, 1–32; Andreas-Holger Maehle, ‘Beyond Professional Self-interest: Medical Ethics and the Disciplinary Function of the General Medical Council of the United Kingdom, 1858–1914’, *Social History of Medicine*, 2018, 33:1, 41–56. The General Medical Council’s Current Guidance, see www.gmc-uk.org/ethical-guidance/ethical-guidance-for-doctors/good-medical-practice/duties-of-a-doctor. Accessed May 2023.

139 Portsmouth History Centre, *The Evening News*, ‘Specialists in the Witness Box: Southsea Doctor’s Defence’ (18 June 1929).

140 ‘Claim Against a Southsea Doctor’.

141 Hutchinson, *Syphilis,* 381.

142 Anne Hanley, ‘“I caught it and yours truly was very sorry for himself”: mapping the emotional worlds of British VD patients’, Anne Hanley and Jessica Meyer (eds), *Patient Voices in Britain, 1840–1948* (Manchester: Manchester University Press, 2021), 299–337.

143 MS.8792/4.

144 Robert James Lee, *Three Lectures Delivered at the Hospital for Sick Children, Great Ormond Street* (London: Baillière, Tindall, & Cox, 1886), 6; RCVD, Appendix to First Report, qq. 9573–83.

145 MS.8792/13; ‘Southsea Doctor Sued: Action by Boy of Nine’.

146 MS.8792/6.

147 ‘Action Against Southsea Doctor: The Concluding Stages’.

148 Hutchinson, *Syphilis*, 496; RCVD, Appendix to First Report, qq. 9573–83.

149 Christabel Pankhurst, *The Great Scourge and How to End It* (London: E. Pankhurst, 1913), p. 72. Original emphasis.

150 MS.8792/13.

151 Harrison, *The Diagnosis and Treatment of Venereal Diseases*, 5–6. See also Edmund T. Burke, *Treatment of Venereal Disease in General Practice* (London: Faber & Gwyer, 1927), 78.

152 MS.8792/3; MS.8792/7.

153 See, for example, F.J. Browne, ‘Venereal Diseases in Pregnancy’, *BMJ*, 1927, 251.

154 Findlay, ‘The Treatment of Syphilis in Infancy and Childhood, 199.

155 Tudor Hart, ‘Going to the Doctor’, 550.

156 Shewry v. Maybury, Letter from Maybury to Eberlie on 12 April 1929, MS.8792/11; MS.8792/10.

157 MS.8792/9; Shewry v. Maybury, Letter from Maybury to Eberlie on 21 November 1928, MS.8792/8.

158 Anon., ‘Charge of Medical Negligence’, 1375–77; Anon., ‘Charge of Professional Negligence’, 1185–87; Anon., ‘Charge of Negligence’, *Lancet*, 1929, 1376; Juror, ‘Shewry v. Maybury: A Juror’s Reflections’, *Lancet*, 1929, 98–99; Anon., ‘Alleged Professional Negligence: Shewry v. Maybury’, *BMJ*, 1929, 167–68.

159 Claire Brock, ‘Risk, Responsibility and Surgery in the 1890s and early 1900s’, *Medical History*, 2013, 57, 322.

160 See, for example, Brock, ‘Risk, Responsibility and Surgery’, 317–37.

161 Anon., ‘Charge of Professional Negligence’, 1186.

162 Anon., ‘Charge of Negligence’, 1376.

163 Anon., ‘Charge of Negligence against a Practitioner’, 1375.

164 For more on the evolution of medical negligence within Common Law and tort, see Price, *Medical Negligence in Victorian Britain*, 2–6.

165 Anon., ‘Charge of Negligence against a Practitioner’, 1375.

166 Ibid.

167 Ibid, 1375.

168 Lex, ‘Shewry v. Maybury’, *Lancet* (20 July 1929), 148.

169 Juror, ‘Shewry v. Maybury: A Juror’s Reflections’, *Lancet,* 1929, 98; W.B. Cosens, ‘Shewry v. Maybury: To the Editor of the Lancet’, *Lancet*, 1929, 147. Original emphasis.

170 A. Knyvett Gordon, ‘Shewry v. Maybury: To the Editor of the Lancet’, *Lancet*, 1929, 147.

171 Portsmouth History Centre, *Hampshire Telegraph and Post*, ‘Doctor Sued by Boy: Case from Southsea’, 1929.

172 Shewry v. Maybury, Proof of Dr Francis Walshe, MS.8792/2.

173 MS.8792/4. Emphasis added.

174 MS.8792/10.

175 MS.8792/7.

176 Ibid.

177 MS.8792/11.

178 Angus H. Ferguson, *Should A Doctor Tell? The Evolution of Medical Confidentiality in Britain* (London: Routledge, 2013), 55–77.

179 MS.8792/3.

180 MS.8792/8.

181 ‘Claim Against a Southsea Doctor’.

182 MS.8792/7.

183 MS.8792/3.

184 MS.8792/5.

185 MS.8792/11; Shewry v. Maybury, Letter from Maybury to Eberlie on 6 June 1929, MS.8792/14.

186 Shewry v. Maybury, Letter from Maybury to Eberlie on 14 May 1929, MS.8792/12.

187 ‘Action Against Southsea Doctor: The Concluding Stages’.

188 Anon., ‘Charge of Medical Negligence against a Practitioner’, 1376.

189 Portsmouth History Centre, *Hampshire Telegraph and Post*, ‘No Judgement: Action Against Southsea Doctor’ (26 July 1929).

190 General Register Office, Deaths in the Subdistrict of Kingston and East Southsea in the County of Portsmouth (1929), Death Certificate for Leslie Eric Shewry.

191 General Register Office, Deaths in the Subdistrict of Kingston and East Southsea in the County of Portsmouth (1929), Death Certificate for Marcus Sidney Shewry.

192 ‘Southsea Doctor Sued: Action by Boy of Nine’.

193 ‘Action Against Southsea Doctor: The Concluding Stages’.

194 Lysander Maybury and household, 1921 Census of England and Wales.

195 Marcus Shewry and household, 1921 Census of England and Wales.

196 Charlotte Oakley and household, 1939 Register.

197 England and Wales Government Probate Death Index (1983), 327; Anon., ‘Dr Lysander Maybury’s Death’, *The Evening News* (30 October 1937). According to the National Archives Currency Converter, £26,632 in 1935 would have been the equivalent of 18,753 days’ wages for a skilled tradesman. www.nationalarchives.gov.uk/currency-converter. Accessed December 2024.

198 Portsmouth History Centre, *The Evening News*, ‘Damages Claim Against Southsea Doctor: Injections Given to a Baby’ (17 June 1929); MS.8792/11.

## Notes

Dr Anne Hanley is a Senior Research Fellow and UKRI Future Leaders Fellow in the Department of Applied Health Sciences at the University of Birmingham. She has written on many aspects of sexual health, including patient care, human experimentation, ethics, medical negligence and harm, health policy, health propaganda, state surveillance, sexual violence, eugenics, infertility, literary representations and oral history. Her research team is mapping Britain’s sexual health histories from the First World War to the AIDS crisis, exploring the complex personal, social, cultural and political factors that shaped people’s health experiences and outcomes.

